# Vitamin B-6 intake is related to physical performance in European older adults: results of the New Dietary Strategies Addressing the Specific Needs of the Elderly Population for Healthy Aging in Europe (NU-AGE) study

**DOI:** 10.1093/ajcn/nqaa368

**Published:** 2021-01-29

**Authors:** Pol Grootswagers, Marco Mensink, Agnes A M Berendsen, Carolien P J Deen, Ido P Kema, Stephan J L Bakker, Aurelia Santoro, Claudio Franceschi, Nathalie Meunier, Corinne Malpuech-Brugère, Agata Bialecka-Debek, Katarzyna Rolf, Susan Fairweather-Tait, Amy Jennings, Edith J M Feskens, Lisette C P G M de Groot

**Affiliations:** TI Food and Nutrition, Wageningen, Netherlands; Division of Human Nutrition and Health, Wageningen University, Wageningen, Netherlands; Division of Human Nutrition and Health, Wageningen University, Wageningen, Netherlands; Division of Human Nutrition and Health, Wageningen University, Wageningen, Netherlands; Department of Laboratory Medicine, University of Groningen, University Medical Center Groningen, Groningen, Netherlands; Department of Laboratory Medicine, University of Groningen, University Medical Center Groningen, Groningen, Netherlands; Department of Internal Medicine, University of Groningen, University Medical Center Groningen, Groningen, Netherlands; Department of Experimental, Diagnostic, and Specialty Medicine and CIG Interdepartmental Center “L. Galvani,” Alma Mater Studiorum, University of Bologna, Bologna, Italy; Department of Experimental, Diagnostic, and Specialty Medicine and CIG Interdepartmental Center “L. Galvani,” Alma Mater Studiorum, University of Bologna, Bologna, Italy; Department of Experimental, Diagnostic, and Specialty Medicine, Alma Mater Studiorum, University of Bologna, Bologna, Italy; Department of Applied Mathematics, Institute of Information Technology, Mathematics, and Mechanics, Lobachevsky State University of Nizhny Novgorod–National Research University, Nizhny Novgorod, Russia; CHU Clermont-Ferrand, CRNH Auvergne, Clermont-Ferrand, France; Université Clermont Auvergne, INRA, UNH, Unité de Nutrition Humaine, CRNH Auvergne, F-63000 Clermont-Ferrand, France; Department of Human Nutrition, Warsaw University of Life Sciences–SGGW, Warsaw, Poland; Department of Human Nutrition, Warsaw University of Life Sciences–SGGW, Warsaw, Poland; Department of Nutrition and Preventive Medicine, Norwich Medical School, University of East Anglia, Norwich, United Kingdom; Department of Nutrition and Preventive Medicine, Norwich Medical School, University of East Anglia, Norwich, United Kingdom; Division of Human Nutrition and Health, Wageningen University, Wageningen, Netherlands; Division of Human Nutrition and Health, Wageningen University, Wageningen, Netherlands

**Keywords:** muscle, physical function, homocysteine, niacin, vitamin B-6, vitamin B-12, folate

## Abstract

**Background:**

Maintenance of high physical performance during aging might be supported by an adequate dietary intake of niacin, vitamins B-6 and B-12, and folate because these B vitamins are involved in multiple processes related to muscle functioning. However, not much is known about the association between dietary intake of these B vitamins and physical performance.

**Objectives:**

The objectives of this study were to investigate the association between dietary intake of niacin, vitamins B-6 and B-12, and folate and physical performance in older adults and to explore mediation by niacin status and homocysteine concentrations.

**Methods:**

We used baseline data from the New Dietary Strategies Addressing the Specific Needs of the Elderly Population for Healthy Aging in Europe (NU-AGE) trial, which included *n *= 1249 healthy older adults (aged 65–79 y) with complete data on dietary intake measured with 7-d food records and questionnaires on vitamin supplement use and physical performance measured with the short physical performance battery and handgrip dynamometry. Associations were assessed by adjusted linear mixed models.

**Results:**

Intake of vitamin B-6 was related to lower chair rise test time [β: –0.033 ± 0.016 s (log); *P* = 0.043]. Vitamin B-6 intake was also significantly associated with handgrip strength, but for this association, a significant interaction effect between vitamin B-6 intake and physical activity level was found. In participants with the lowest level of physical activity, higher intake of vitamin B-6 tended to be associated with greater handgrip strength (β: 1.5 ± 0.8 kg; *P* = 0.051), whereas in participants in the highest quartile of physical activity, higher intake was associated with lower handgrip strength (β: –1.4 ± 0.7 kg; *P* = 0.041). No evidence was found for an association between intake of niacin, vitamin B-12, or folate and physical performance or for mediation by niacin status or homocysteine concentrations.

**Conclusions:**

Vitamin B-6 intake was associated with better chair rise test time in a population of European healthy older adults and also with greater handgrip strength in participants with low physical activity only. Homocysteine concentrations did not mediate these associations. The NU-AGE trial was registered at clinicaltrials.gov as NCT01754012.

## Introduction

Life expectancy has been steadily increasing during the past 200 y (1). However, the extra years of life are often lived in poor health. To age more healthily, older adults need to maintain a high level of physical performance in daily life because this is related to increased independence and a higher quality of life (2). Furthermore, high physical performance reduces chances of hospitalization (3), cognitive decline (4), and mortality (5). B vitamins have been suggested to protect against age-related physical decline (6). However, the exact role of B vitamin intake in relation to physical performance in older adults is unknown, and evidence for associations is scarce.

B vitamins play an important role in at least 3 mechanisms related to physical performance. First, intake of vitamins B-6, B-12, and folate reduces homocysteine concentrations (7). High concentrations of homocysteine induce inflammation and decrease neurological functioning, which might lead to decreased physical performance (8). This is in line with multiple studies that have shown that homocysteine concentrations are inversely associated with quadriceps strength (9), chair rise test performance (10), and gait speed (10–12). Second, niacin, vitamins B-6 and B-12, and folate contribute to the functioning of mitochondria (13). Mitochondrial functioning is impaired by elevated homocysteine concentrations (14). Thus, lowering intracellular homocysteine concentrations by adequate vitamin B-6, vitamin B-12, and folate intakes may improve mitochondrial functioning, which can benefit physical performance (15). In addition, dietary intake of niacin (and its precursor tryptophan) can lead to increased concentrations of NAD (16). Increasing NAD concentrations by pharmacological agents has been shown to be an effective intervention to improve mitochondrial functioning in humans (17). In mice, long-term administration of an NAD precursor reduced age-related physiological decline (18). Third, B vitamins may preserve muscle quality via their antioxidant properties (19) and by improving neuromuscular functioning (6). Taken together, B vitamins may contribute to physical performance via suppressing homocysteine concentrations, improving mitochondrial functioning, and preserving muscle quality.

Despite these indications of an important role of B vitamin intake in relation to physical performance, only 2 observational studies have investigated this association, within the same cohort. These studies showed that adequate intake of vitamin B-6, vitamin B-12, and folate led to lower risk of impaired mobility (20) and frailty (21). In these 2 studies, dietary intake was assessed via the dietary history method, and level of physical performance was self-reported. In this article, we report the results of our investigation of the association between objectively measured physical performance, as measured by handgrip strength, walking speed, and chair rise test, and dietary intake, as measured by detailed 7-d food records, in a large European cohort of older adults. In addition, we explore potential mediation by homocysteine concentrations and niacin status.

## Methods

### Study design

We used baseline data from the New Dietary Strategies Addressing the Specific Needs of the Elderly Population for Healthy Aging in Europe (NU-AGE) study for this analysis. The NU-AGE study is a dietary intervention trial that was performed in 5 centers in different European countries (France, Italy, the Netherlands, Poland, and the United Kingdom). It is registered at clinicaltrials.gov under NCT01754012. The study design and participant recruitment have been described in detail elsewhere (22, 23). In summary, 2668 apparently healthy, community-dwelling older adults (aged 65–79 y) were recruited for the trial, of which 1518 attended the screening and 1296 eligible participants were included (for flowchart, see **Supplemental Figure 1**). The primary outcome of the NU-AGE trial was the change in C-reactive protein after 1 y of dietary intervention. For this study, we used data obtained before the start of the dietary intervention of 1249 participants who completed the baseline dietary intake assessment.

### Dietary intake

Dietary intake was assessed through 7-d food records. Before the measurement period, participants were trained in describing foods, portion sizes, preparation methods, complex recipes, and overall level of detail (24, 25). Participants reported portion sizes in relation to national household measures, pictures, or as measured in grams or millimeters. Dietitians or research nutritionists, who were trained in a standardized way to reduce between-center variation, reviewed the level of detail of the completed food records during a home visit (in the Netherlands and United Kingdom) or at the research center (Poland, Italy, and France). Food records were coded according to standardized coding procedures. Nutrient content was calculated by using local food composition tables [NEVO 2011 in the Netherlands (26), McCance and Widdowson in the UK (27), INRAN and BDA in Italy (28, 29), NFNI in Poland (30), and CIQUAL French food composition table in France (31)]. Participants from the French center were excluded for vitamin B-12 analyses due to unreliable intake data for this vitamin. Supplementary vitamin B intake was assessed by means of an additional questionnaire, and participants using vitamin B–containing supplements were identified and excluded from a sensitivity analysis. Niacin equivalents were calculated as follows: niacin (mg) + [tryptophan (mg)/60]. Tryptophan intake was estimated from total protein intake by assuming an average abundance of 1.1% (32).

### Physical performance

Physical performance was measured at all centers by the Short Physical Performance Battery (SPPB) and handgrip strength. The SPPB was performed following the protocol described by Guralnik et al. (33) and consisted of a 2.44-m usual gait speed test, a balance test in 3 foot positions of increasing difficulty, and a 5-time chair rise test. Handgrip strength was measured by dynamometry (Scandidact Smedley's Hand Dynamometer) to the nearest 0.1 kg, in standing position with the arm flexed at 90°. For each hand, the maximum value of 3 repetitions was used for analysis. The measurements of physical performance were standardized over the 5 centers. The standardization was done by joint training sessions in Warsaw and Wageningen, where representatives from all 5 centers were present. Physical activity was expressed in Physical Activity Scale for Elderly (PASE) questionnaire-based PASE scores (34). These questionnaires were self-administered and checked by a researcher.

### Biochemical analyses

Urine samples were collected during day 7 of the dietary intake assessment, which was 1 d prior to physical performance assessment. Participants were instructed to discard the first urine of the day and to collect all following urine, including the first urine of the second day, in standard containers that contained 2.7 mL of 1% sodium azide solution. Total urine volume was recorded, and urine was aliquoted in cryovials and stored at –80°C until analysis. Urinary *N*^1^-methylnicotinamide (NMN) and *N*-methyl-2-pyridone-carboxamide (2-Pyr) concentrations were measured at University Medical Centrum Groningen, the Netherlands, by the validated LC (Luna HILIC column; Phenomenex) isotope dilution MS/MS method (Quattro Premier; Waters), as described previously (35). The sum of the 2 metabolite concentrations was multiplied by total 24-h urine volume to obtain 24-h urinary niacin metabolite excretion, which is considered a biomarker of niacin status (36).

Plasma homocysteine concentrations were assessed by the enzymatic method (Olympus AU400 chemistry analyzer; Beckman) in the Nigrisoli hospital in Bologna, Italy, as described previously (37).

### Statistical analysis

For descriptive purposes, energy-adjusted tertiles of the 4 vitamins (niacin, B-6, B-12, and folate) were created using the residual method (38). Descriptive statistics are presented as means ± SDs for normally distributed values and as medians (25th–75th percentile) for skewed variables. Skewed outcome variables (walk time and chair rise test time) were log transformed. All analyses were performed using SAS software version 9.4 (SAS Institute).

Associations between intake of B vitamins and measures of physical performance were assessed via mixed linear models. Continuous, unadjusted measures of the B vitamins were used as exposure, and energy adjustment was performed via adding energy intake to the model. Possible effect modification by physical activity level was tested by adding the interaction term exposure*physical activity to the model. The interaction model was used when the interaction model showed an improved model fit over the model without interaction term, as concluded from likelihood ratio tests using maximum likelihood estimation (39).

Three models of increasing complexity were built to adjust for confounding factors. The first model only adjusted for age and sex. The second model additionally adjusted for energy intake, drinking status (non, light, or heavy), smoking status (never, former, or current), education level (low, medium, or high), and physical activity level. The third model additionally adjusted for protein intake and study center. Adjustment for study center was performed by including study center as a random factor in a random intercept model.

Additional analyses were done to test for a possible mediating effect of niacin metabolites in the niacin–physical function relation and for homocysteine concentrations in the vitamin B-6, vitamin B-12, folate–physical performance relation. We calculated a normalized combined vitamin score for vitamin B-6, vitamin B-12, and folate by dividing all values by the mean intake of the specific vitamin and summing up those values. For example, if a participant had an intake around the mean for vitamin B-6, vitamin B-12, and folate, the combined score for this participant was ∼3. We followed the mediation analysis method described by Preacher and Hayes (40) and concluded that there was no evidence for mediation when the potential mediator was not associated with either exposure or outcome. Associations between mediator and exposure or outcome were performed by using the fully adjusted models.

We performed 2 sensitivity analyses. The first sensitivity analysis was performed to assess the influence of possible under- or overreporters of energy intake. For this analysis, we excluded participants with a daily average energy intake outside the 500- to 4000-kcal range and participants with an energy intake to basal metabolic rate [estimated by Schofield's equation (41)] ratio outside the 0.8—2.66 range. In the newly created data set, we reran the programs of the second and third models and compared outputs. The second sensitivity analysis excluded participants using vitamin B–containing supplements.

## Results

Participants (*n *= 1249) had a mean age of 71 ± 4 y, and 56% were women (**Table 1**). The study population had a high level of physical functioning, with a median SPPB score of 12. Measures of physical performance were similar over the 5 centers, except for high handgrip strength in participants from the United Kingdom, even despite the higher female proportion in this center. The UK participants also showed higher scores on the PASE questionnaire and had the highest intakes of niacin, vitamin B-6, and vitamin B-12. Participants from France had the highest daily average energy and protein intake but the lowest BMI. On the other hand, participants from Italy had the lowest average daily intake of energy, protein, and vitamin B-12 and the lowest score on the PASE questionnaire. In the Netherlands, participants had the lowest intake levels of 3 out of 4 B vitamins: niacin, vitamin B-6, and folate.

**TABLE 1:** Baseline characteristics of the total study sample and per study center^1^

	Total (*n* = 1249)	Italy (*n* = 273)	United Kingdom (*n* = 272)	The Netherlands (*n* = 252)	Poland (*n* = 251)	France (*n* = 201)
Gender, % female	56	52	64	56	57	50
Age, y	70.9 ± 4.0	71.7 ± 3.9	70.1 ± 4.0	71.0 ± 4.0	71.4 ± 3.8	71.2 ± 3.9
Height, cm	166 ± 9	164 ± 10	166 ± 9	169 ± 8	164 ± 9	166 ± 9
Weight, kg	73.6 ± 13.4	73.0 ± 12.6	74.0 ± 13.7	74.6 ± 13.2	75.3 ± 14.2	70.1 ± 12.6
BMI, kg/m^2^	26.7 ± 4.0	27.2 ± 3.9	26.8 ± 4.1	26.0 ± 3.6	28.0 ± 4.2	25.4 ± 3.5
Diabetes, %	5	6	3	4	8	4
Hypertension, %	41	49	29	33	61	33
Hypercholesterolemia, %	33	48	22	25	38	31
Osteoporosis, %	12	12	4	10	20	13
Smoker, % never/former/current	53/42/5	44/49/7	60/38/2	51/46/3	49/44/7	66/31/3
Drinker,^2^ % non/light/heavy	17/71/11	31/53/16	10/82/9	13/72/15	21/55/24	10/71/18
Education,^3^ low/medium/high	13/52/35	39/39/22	1/83/16	13/56/31	1/28/71	11/53/36
PASE score	133 ± 55	113 ± 50	149 ± 53	137 ± 53	131 ± 62	133 ± 52
Homocysteine, µmol/L	12.6 ± 4.3	14.5 ± 4.2	12.8 ± 3.8	11.1 ± 3.0	12.5 ± 5.2	11.9 ± 4.2
Niacin intake,^4^ mg/d	17.0 (13.9–20.7)	17.2 (13.7–20.9)	18.4 (15.6–22.2)	15.7 (13.3–18.6)	17.0 (13.4–21.3)	16.5 (13.7-20.0)
Vitamin B-6 intake, mg/d	1.8 ± 0.6	1.6 ± 0.5	2.0 ± 0.6	1.6 ± 0.5	2.0 ± 0.7	1.7 ± 0.4
Folate intake, μg/d	290 ± 97	271 ± 106	309 ± 96	255 ± 71	297 ± 98	323 ± 93
Vitamin B-12 intake,^4^ μg/d)	4.3 (3.0–6.3)	2.7 (1.9–4.1)	5.9 (4.5–7.8)	4.3 (3.4–5.6)	4.1 (2.9–6.3)	NA
Protein intake, g/d	75 ± 18	67 ± 14	77 ± 16	76 ± 16	77 ± 22	81 ± 17
Energy intake, kcal/d	1864 ± 445	1711 ± 381	1895 ± 382	1908 ± 411	1822 ± 512	2026 ± 484
B supplement users, %	19	15	20	26	21	11
Dominant handgrip strength, kg	31.5 ± 9.5	30.9 ± 9.7	34.5 ± 9.0	30.5 ± 9.2	30.3 ± 9.8	31.0 ± 8.8
Total SPPB score^4^	12 (11–12)	12 (11–12)	12 (11–12)	12 (11–12)	12 (11–12)	12 (11–12)
Repeated chair rise time, s	10.3 ± 2.8	10.3 ± 2.9	10.6 ± 2.8	10.0 ± 2.7	10.2 ± 2.7	10.3 ± 2.6
Time to walk 2.44 m, s	2.4 ± 0.6	2.3 ± 0.6	2.3 ± 0.6	2.5 ± 0.5	2.5 ± 0.6	2.6 ± 0.5

1 Values are means ± SDs unless stated otherwise. NA, not applicable; PASE, Physical Activity Scale for the Elderly; SPPB, Short Physical Performance Battery.

2 Light drinker, 1–14 units per week; heavy drinker, ≥14 units per week.

3 Low, 0–8 y; medium, 9–13 y, high, ≥14 y.

4 Median (IQR).

Characteristics of the participants per energy-adjusted tertiles of niacin, vitamin B-6, folate, or vitamin B-12 intake are presented in **Table 2**. Apart from folate intake, the gender distribution was similar in all 3 tertiles. For folate intake, a higher proportion of men was observed in the first tertile compared with the other tertiles. Participants with higher folate intake were more likely to have a lower body weight and handgrip strength, whereas participants with higher intake of niacin, vitamin B-6, and vitamin B-12 were more likely to have higher body weight and handgrip strength. Other measures of physical performance were similar in the different tertiles of all the vitamins. Participants with lower intakes of vitamin B-6, vitamin B-12, and folate were more likely to have fewer years of education. Low-folate consumers were more often heavy drinkers (≥14 units per week), whereas low-B-12 consumers were more often nondrinkers. Participants with higher intake of any of the 4 vitamins consumed more protein and more of the other 3 B vitamins. Significant correlations were observed for the 6 pairs of B vitamins, ranging from *r* = 0.22 for the correlation between intake of niacin and vitamin B-12 to *r* = 0.61 for the correlation between intake of vitamin B-6 and folate. Participants with a high intake of folate or vitamin B-12 were more likely to have lower homocysteine concentrations.

**TABLE 2:** Baseline characteristics per tertile of B vitamin intake^1^

	Niacin			Vitamin B-6			Folate			Vitamin B-12		
	Tertile 1	Tertile 2	Tertile 3	Tertile 1	Tertile 2	Tertile 3	Tertile 1	Tertile 2	Tertile 3	Tertile 1	Tertile 2	Tertile 3
*n*	415	417	417	416	417	416	416	417	416	349	350	349
Gender, % female	57	60	52	55	57	56	47	62	59	54	61	57
Age, y	71 ± 4	71 ± 4	71 ± 4	71 ± 4	71 ± 4	71 ± 4	71 ± 4	71 ± 4	71 ± 4	71 ± 4	71 ± 4	71 ± 4
Height, cm	166 ± 9	165 ± 9	166 ± 9	166 ± 9	165 ± 10	166 ± 9	167 ± 9	165 ± 9	165 ± 9	165 ± 9	165 ± 9	166 ± 9
Weight, kg	71.9 ± 12.5	73.4 ± 13.8	75.4 ± 13.7	72.8 ± 12.8	73.9 ± 13.8	74.0 ± 13.6	75.8 ± 12.9	72.8 ± 13.8	72.1 ± 13.3	73.4 ± 12.8	73.0 ± 13.3	76.2 ± 14.1
BMI, kg/m^2^	26.1 ± 3.5	26.8 ± 4.1	27.4 ± 4.1	26.3 ± 3.6	27.0 ± 4.0	26.9 ± 4.2	27.0 ± 3.6	26.8 ± 4.2	26.4 ± 3.9	26.9 ± 3.8	26.6 ± 3.9	27.5 ± 4.2
Diabetes, %	4	4	7	3	5	7	7	2	6	7	4	4
Hypertension, %	35	42	46	36	44	44	44	41	39	44	40	44
Hypercholesterolemia, %	29	32	38	31	31	37	33	32	34	36	31	33
Osteoporosis, %	16	10	9	14	11	10	10	14	12	14	13	8
Smoker, % never/former/current	60/37/3	53/44/3	47/46/7	55/40/2	52/44/4	53/43/4	47/46/7	59/38/3	54/42/4	50/43/7	49/46/4	53/44/3
Drinker,^2^ % non/light/heavy	19/63/18	18/68/14	15/67/17	17/63/21	16/69/14	19/67/14	16/64/20	17/69/14	20/66/14	29/55/16	14/70/17	14/71/15
Education,^3^ low/medium/high	13/52/35	15/48/37	12/56/32	18/46/36	15/53/32	8/57/35	18/52/30	12/52/36	11/52/37	23/46/31	11/51/37	7/58/34
PASE score	134 ± 54	131 ± 56	133 ± 56	133 ± 58	129 ± 53	137 ± 55	131 ± 56	133 ± 55	134 ± 56	124 ± 59	135 ± 50	139 ± 58
Homocysteine, µmol/L	12.9 ± 4.7	12.5 ± 4.2	12.5 ± 3.8	12.8 ± 4.3	12.6 ± 4.6	12.4 ± 3.8	13.1 ± 4.4	12.3 ± 4.3	12.4 ± 3.9	13.9 ± 4.7	12.1 ± 4.0	12.2 ± 3.7
Niacin intake,^4^ mg/d	13.1 (11.1–14.9)	16.7 (14.9–18.6)	22.1 (19.7–25.8)	14.5 (12.1–17.7)	16.5 (14.0–19.6)	20.2 (17.1–24.4)	16.0 (13.1–19.2)	16.1 (13.4–19.2)	19.3 (15.7–23.4)	15.9 (12.9–19.5)	16.4 (13.5–19.9)	18.6 (15.7–-22.3)
Vitamin B-6 intake, mg/d	1.5 ± 0.4	1.7 ± 0.4	2.2 ± 0.6	1.4 ± 0.3	1.7 ± 0.3	2.3 ± 0.6	1.6 ± 0.5	1.7 ± 0.5	2.1 ± 0.6	1.7 ± 0.5	1.8 ± 0.6	2.0 ± 0.6
Folate intake, μg/d	264 ± 82	281 ± 91	323 ± 106	248 ± 77	278 ± 82	342 ± 104	208 ± 49	274 ± 43	387 ± 87	272 ± 102	271 ± 78	307 ± 103
Vitamin B-12 intake,^4^ μg/d	3.8 (2.6–5.2)	4.2 (2.9–5.9)	4.9 (3.5–8.0)	3.8 (2.5–5.2)	4.2 (3.0–6.0)	4.9 (3.5–7.6)	4.0 (2.6–5.7)	4.3 (3.1–6.1)	4.7 (3.1–7.5)	2.5 (1.9–3.2)	4.2 (3.6–4.8)	7.5 (6.1–10.0)
Protein intake, g/d	71 ± 16	74 ± 15	81 ± 20	71 ± 16	74 ± 16	81 ± 20	73 ± 17	74 ± 16	79 ± 19	69 ± 17	73 ± 15	81 ± 19
Energy intake, kcal/d	1901 ± 457	1815 ± 414	1876 ± 458	1912 ± 461	1815 ± 417	1865 ± 450	1882 ± 463	1833 ± 419	1877 ± 450	1849 ± 448	1777 ± 398	1873 ± 437
B supplement users, %	21	20	18	18	18	22	14	22	21	18	24	20
Dominant handgrip strength, kg	30.8 ± 9.3	31.2 ± 9.4	32.5 ± 9.6	30.8 ± 9.5	31.4 ± 9.3	32.2 ± 9.6	32.6 ± 9.7	30.8 ± 9.0	31.1 ± 9.6	31.0 ± 9.8	30.7 ± 9.1	33.1 ± 9.7
Total SPPB score^4^	12 (11–12)	12 (11–12)	12 (11–12)	12 (11–12)	12 (11–12)	12 (11–12)	12 (11–12)	12 (11–12)	12 (11–12)	12 (11–12)	12 (11–12)	12 (11–12)
Repeated chair rise time, s	10.3 ± 2.9	10.3 ± 2.5	10.2 ± 2.8	10.2 ± 2.5	10.5 ± 3.1	10.2 ± 2.7	10.3 ± 2.6	10.4 ± 3.0	10.2 ± 2.7	10.2 ± 2.9	10.4 ± 2.7	10.2 ± 2.7
Time to walk 2.44 m, s	2.5 ± 0.6	2.5 ± 0.6	2.4 ± 0.5	2.5 ± 0.5	2.5 ± 0.6	2.4 ± 0.5	2.4 ± 0.5	2.5 ± 0.6	2.4 ± 0.4	2.4 ± 0.6	2.4 ± 0.6	2.4 ± 0.6

1 Values are means ± SDs unless stated otherwise. PASE, Physical Activity Scale for the Elderly; SPPB, Short Physical Performance Battery.

2 Light drinker, 1–14 units per week; heavy drinker, ≥14 units per week.

3 Low, 0–8 y; medium, 9–13 y, high, ≥14 y.

4 Median (IQR).

**Table 3** presents the associations between intake of the 4 B vitamins and measures of physical performance. Intake of vitamin B-6 was related to improved chair rise test time [β: –0.033 ± 0.016 s (log); *P* = 0.043], translating to a 3.2% lower chair rise time per milligram higher vitamin B-6 intake. Niacin and vitamin B-6 intakes were significantly and positively associated with handgrip strength. However, for these associations, there was a significant interaction between vitamin intake and physical activity level. In participants with the lowest level of physical activity, intake of vitamin B-6 tended to be positively associated with handgrip strength (**Table 4**; β: 1.5 ± 0.8 kg; *P* = 0.051], whereas the associations were negative in participants with the highest level of physical activity (β: –1.4 ± 0.7 kg; *P* = 0.041; interaction effect: *P* = 0.003). There were no significant associations between niacin intake and handgrip strength at any physical activity level. Participants in the highest physical activity quartile had a higher handgrip strength compared with those in the lowest physical activity quartile (Δ: 4.4 ± 0.7 kg; *P* < 0.0001; **Supplemental Table 1**). They also had a higher intake level of total energy (Δ: 170 ± 37 kcal; *P* < 0.0001), protein (Δ: 6 ± 1 g; *P* < 0.0001), and vitamin B-6 (Δ: 0.2 ± 0.0 mg; *P* < 0.001) but not niacin (Δ: 0.5 ± 0.5 mg; *P* = 0.309).

**TABLE 3:** Association between intake of the 4 different vitamins and measures of physical functioning^1^

	Handgrip strength (*n* = 1237)				Walk time (log) (*n* = 1234)		Repeated chair rise time (log) (*n* = 1228)	
	β exposure	*P* value	β interaction	*P* value	β exposure	*P* value	β exposure	*P* value
			Exposure*PASE score					
Niacin								
Model 1	0.07 ± 0.02	0.004			–0.002 ± 0.001	0.053	–0.001 ± 0.001	0.578
Model 2	0.17 ± 0.06	0.009	–0.001 ± 0.000	0.057	–0.002 ± 0.001	0.062	–0.0004 ± 0.001	0.743
Model 3	0.15 ± 0.06	0.014	–0.001 ± 0.000	0.008	0.0001 ± 0.001	0.964	–0.001 ± 0.001	0.580
Vitamin B-6								
Model 1	1.42 ± 0.31	<0.0001			–0.039 ± 0.011	<0.001	–0.024 ± 0.013	0.058
Model 2	2.71 ± 0.76	<0.001	–0.012 ± 0.005	0.015	–0.040 ± 0.012	0.001	–0.016 ± 0.014	0.261
Model 3	1.76 ± 0.74	0.018	–0.014 ± 0.005	0.003	–0.025 ± 0.014	0.073	–0.033 ± 0.016	0.043
Folate								
Model 1	0.003 ± 0.002	0.083			–0.0001 ± 0.0001	0.024	–0.0001 ± 0.0001	0.130
Model 2	0.001 ± 0.002	0.702			–0.0001 ± 0.0001	0.062	–0.0001 ± 0.0001	0.613
Model 3	–0.002 ± 0.002	0.235			–0.0001 ± 0.0001	0.092	–0.0001 ± 0.0001	0.292
Vitamin B-12								
Model 1	0.159 ± 0.05	0.001			–0.002 ± 0.002	0.213	–0.002 ± 0.002	0.340
Model 2	0.114 ± 0.05	0.020			–0.001 ± 0.002	0.442	–0.0004 ± 0.002	0.844
Model 3	–0.004 ± 0.05	0.936			–0.0014 ± 0.002	0.459	–0.003 ± 0.002	0.247

1 Model 2: additional adjustment for energy intake, drinking status, smoking status, education level, and physical activity level. Model 3: additional adjustment for study center and protein intake. PASE, Physical Activity Scale for the Elderly.

**TABLE 4:** Association between intake of niacin or vitamin B-6 and handgrip strength over the 4 quartiles of physical activity^1^

	Q1 (*n* = 311) Median PASE score: 72; range: 5–94			Q2 (*n* = 311) Median PASE score: 112; range: 94–128			Q3 (*n* = 317) Median PASE score: 146; range: 128–167			Q4 (*n* = 306) Median PASE score: 195; range: 167–548		
	Intercept	β exposure	*P* value	Intercept	β exposure	*P* value	Intercept	β exposure	*P* value	Intercept	β exposure	*P* value
Niacin–handgrip strength	56.0 ± 7.1	0.07 ± 0.05	0.181	58.9 ± 8.6	–0.02 ± 0.05	0.638	61.5 ± 8.1	0.02 ± 0.05	0.657	57.6 ± 7.4	–0.05 ± 0.07	0.481
Vitamin B-6–handgrip strength	56.4 ± 7.1	1.5 ± 0.8	0.051	59.4 ± 8.7	0.2 ± 0.8	0.787	61.3 ± 8.1	–0.2 ± 0.8	0.783	56.8 ± 7.4	–1.4 ± 0.7	0.041

1 Data are from the model adjusted for age, sex, energy intake, drinking status, smoking status, education level, physical activity level, study center, and protein intake. PASE, Physical Activity Scale for the Elderly.

Results of mediation analyses are presented in **Supplemental Table 2**. Combined normalized intake of vitamin B-6, vitamin B-12, and folate was related to lower homocysteine concentrations in the fully adjusted model (*P* = 0.003). A higher intake of each of the vitamins was related to lower homocysteine concentrations (β vitamin B-6: –0.46 ± 0.26 µmol/L, *P* = 0.084; β folate: –0.003 ± 0.001 µmol/L, *P* = 0.056; and β vitamin B-12: –0.08 ± 0.03 µmol/L, *P* = 0.024). However, homocysteine concentrations were not related to handgrip strength, walk time, or repeated chair rise test time (*P *> 0.10 for all outcomes).

Intake of niacin equivalents was associated with total urinary niacin metabolites (β: 0.53 ± 0.26 µmol/L; *P* = 0.045). Intake of niacin by itself was not associated with the niacin metabolites (β: 0.48 ± 0.33 µmol/L; *P* = 0.147). The sum of the urinary metabolites was not associated with handgrip strength, walking time, or chair rise test (*P *> 0.30 for all outcomes).

Excluding energy misreporters (*n* = 28 underreporters, *n *= 0 overreporters) resulted in an attenuated, but still significant, association between vitamin B-6 intake and handgrip strength (with effect modification by physical activity level) and attenuation to insignificance for the association between vitamin B-6 intake and chair rise test time [β: –0.03 ± 0.02 s (log); *P* = 0.053; **Supplemental Table 3**]. Excluding 240 participants who used B vitamin–containing supplements resulted in significant associations between intake of vitamin B-6 and improved walk time [β: –0.04 ± 0.02 s (log); *P* = 0.011] and between intake of folate and walk time [β: –0.0002 ± 0.0001 s (log); *P* = 0.012]. The results of this analysis are presented in **Supplemental Table 4**.

## Discussion

This study aimed to assess whether dietary intake of niacin, vitamin B-6, vitamin B-12, and folate is associated with physical performance in healthy European older adults. We found that higher intakes of vitamin B-6 were associated with a better chair rise test performance and that vitamin B-6 intake was associated with improved handgrip strength in participants with low physical activity levels.

The association between vitamin B-6 and handgrip strength was observed in participants in the lowest quartile of physical activity. The median PASE score of these participants was 72, which is considerably lower than recommended levels. A PASE score of 130 is recommended because this predicts favorable body composition measures such as waist circumference (42). To increase PASE score from 72 to 130, one would have to increase activity by as much as 2 additional hours of moderate to strenuous physical activities on at least 5 d per week (42). High levels of physical activity might increase the requirements of vitamin B-6 (43). Vitamin B-6 is needed for glycogen breakdown and amino acid metabolism, 2 metabolic pathways that are upregulated by exercise (44), and that may explain the observed interaction.

In contrast, the relation between vitamin B-6 and chair rise test performance did not vary by physical activity levels. This discrepancy is interesting because handgrip strength and chair rise test both reflect limb muscle strength (45), and upper and lower limb strength are strongly correlated (46). However, because the chair rise test is timed, it reflects muscle power, whereas handgrip strength reflects muscle strength, which might underlie the difference. Future studies should explore the relation between B-6 intake, status, and physical performance over different levels of physical activity.

Similar to our findings, a role for vitamin B-6 intake in maintaining physical performance has previously been reported 3 times (20, 21, 47). First, Struijk et al. (20) showed that a higher intake of vitamin B-6 was associated with a lower risk of impaired mobility in *n *= 1630 Spanish older adults. Participants in the highest tertile of vitamin B-6 intake had a significantly reduced risk of developing impaired mobility compared with participants in the lowest tertile. However, the authors found no evidence for an association between vitamin B-6 intake and physical performance. Note that in their study, physical performance was assessed by questionnaires, which may have introduced bias. Similar to our findings, Struijk et al. (20) did not find associations between vitamin B-12 and folate intake with measures of physical performance. A second study, in the same Spanish cohort, found that participants in the lowest tertiles for vitamin B-6 and folate intake had more than double the risk of developing frailty compared with participants in the highest tertiles (21). Low intakes of vitamin B-12 did not increase frailty risk in that study. Finally, by applying mixed graphical models to merged data from different cohorts of Dutch older adults (*n *= 662), Behrouzi et al. (47) identified direct associations between vitamin B-6 and B-12 intake and physical performance.

Combined intake of vitamin B-6, vitamin B-12, and folate was related to lower concentrations of homocysteine, but we did not observe any relation between homocysteine concentrations and physical performance. This observation is in contrast with previous studies in older adults, which showed that high homocysteine concentrations negatively correlate with physical performance (9–11) while adjusting for similar covariates as in our models. Homocysteine concentrations were lower than (9), higher than (10), or comparable to (11) homocysteine concentrations in our study, and physical performance was similar in 2 of the 3 studies [walk speed of 1.0 ± 0.2 m/s (9) and 1.1 ± 0.2 m/s (11) compared to 1.0 ± 0.2 m/s in our study] and lower in the third study [mean SPPB score of 7.4 ± 3.2 (10) compared to 11.3 ± 1.1 in our study]. Possibly, a prolonged homocysteine reduction could still support physical performance maintenance in our population.

In addition to lowering homocysteine concentrations, vitamin B-6 plays a role in the metabolism of many amino acids, neurotransmitters, and fatty acids (48). Vitamin B-6 status is related to plasma concentrations of PUFAs, and short-term dietary vitamin B-6 restriction can already decrease plasma concentrations of especially n–3 fatty acids (49). Moreover, dietary B-6 restriction leads to lower plasma creatine concentrations (50). Both n–3 fatty acids and creatine concentrations are related to measures of physical performance (51, 52). Therefore, the role of vitamin B-6 in the metabolism of fatty acids and amino acids that are important for an optimal physical function may underlie the associations found in the current study.

The main food sources of vitamin B-6 intake in European adults are meat, dairy products, vegetables, fruits, and potatoes. Intakes of these sources differed over the 5 countries (23). The high contribution of animal foods to vitamin B-6 intake should be noted regarding our observed associations because animal foods also contribute to increased intake of high-quality protein. High-quality protein intake is known to improve muscle protein synthesis and might contribute to physical performance (53). Protein intake was 81 g/d in the highest vitamin B-6 tertile and 71.3 g/d in the lowest tertile. We adjusted our final models for total protein intake to overcome this issue.

Niacin intake was associated with handgrip strength but differed across levels of physical activity. We did observe a significant positive association between dietary intake of niacin equivalents and 24-h urinary excretion of NMN and 2-Pyr, which is in line with previous findings (36). Niacin intake by itself was not related to the urinary niacin metabolites. This finding supports the observation that tryptophan intake is quantitatively more important for NAD^+^ availability than niacin intake (54). Although our data suggest that niacin intake plays a role in physical performance, we are not able to conclude it from our findings.

Interestingly, the exclusion of vitamin B supplement users revealed that intakes of vitamin B-6 and folate were related to a better walk test performance. Possibly, the dietary form of these vitamins exerts larger beneficial results on physical performance compared with the different form that is used in supplements (55, 56). Excluding possible energy underreports resulted in attenuation of the association between vitamin B-6 intake and physical performance.

This study has some limitations. First, due to its cross-sectional nature, we were not able to draw any conclusions regarding causality. Second, to avoid losing statistical power in testing our prespecified contrasts, we did not correct for multiplicity in our analyses (57, 58). Third, physical performance was very good in this cohort. We hypothesize that the associations that we found in this high-functional cohort with little interindividual variance might be larger when studied in a more fragile population. Our sample size was large, and we had excellent exposure and outcome assessment. In addition, outcome measurements were well standardized over the centers. Finally, a strength is that in our models we adjusted for many important confounders, including protein intake.

In conclusion, in this large population of European older adults with a high level of physical performance, we found positive associations between vitamin B-6 and chair rise test performance in the full population and between vitamin B-6 intake and handgrip strength in participants with low physical activity. Homocysteine concentrations did not mediate these associations. Vitamin B-6 might be of added value in preventing age-related decline in physical performance, especially in cases in which increasing physical activity is not feasible.

## Supplementary Material

nqaa368_Supplemental_File

## Data Availability

Data described in the manuscript, code book, and analytic code will be made available upon request pending application and approval.

## Notes

This work was supported by the TIFN research program Mitochondrial Health (ALWTF.2015.5). The NU-AGE trial was supported by the European Union's Seventh Framework Program (266486). The funders had no role in the design and implementation of the study nor in data analysis and interpretation.

Supplemental Tables 1–4 and Supplemental Figure 1 are available from the “Supplementary data” link in the online posting of the article and from the same link in the online table of contents at https://academic.oup.com/ajcn/.

Abbreviations used: NMN, *N***1**-methylnicotinamide; NU-AGE, New Dietary Strategies Addressing the Specific Needs of the Elderly Population for Healthy Aging in Europe; PASE, Physical Activity Scale for Elderly; SPPB, Short Physical Performance Battery; 2-Pyr, *N*-methyl-2-pyridone-carboxamide.
