# Supplementary material for: Vitamin B-6 intake is related to physical performance in European older adults: results of the New Dietary Strategies Addressing the Specific Needs of the Elderly Population for Healthy Aging in Europe (NU-AGE) study
Source: Am J Clin Nutr. 2021 Jan 29;113(4):781–9. doi: 10.1093/ajcn/nqaa368 (PMC8024000; doi:10.1093/ajcn/nqaa368)
Supplement: nqaa368_Supplemental_File [file nqaa368_supplemental_file.docx]

On-line Supplementary Material - Vitamin B-6 intake is related to physical performance in European older adults - the NU-AGE study
*Grootswagers, P. et al.*

| **Supplemental Table 1.** Differences between physical activity quartile 1 and 4. | | | | |
| --- | --- | --- | --- | --- |
|  | Physical activity quartile 1 (*n*=311) | Physical activity quartile 4 (*n*=306) | Difference | P-value |
| Energy intake (kcal/d) | 1805 (430) | 1974 (478) | 170 ± 37 | <.0001 |
| Protein intake (g/d) | 73 (17) | 79 (19) | 6 ± 1 | <.0001 |
| Vitamin B3 intake (mg/d) | 17.8 (7.0) | 18.4 (6.0) | 0.5 ± 0.5 | 0.309 |
| Vitamin B6 intake (mg/d) | 1.7 (0.5) | 1.9 (0.7) | 0.2 ± 0.05 | <.001 |
| Folate intake (µg/d) | 283 (94) | 299 (95) | 17 ± 8 | 0.03 |
| Vitamin B12 intake (µg/d) | 5.0 (4.1) | 5.9 (5.4) | 0.9 ± 0.4 | 0.007 |
| Handgrip strength (kg) | 30 (9) | 34 (9) | 4.4 ± 0.7 | <.0001 |

| **Supplemental Table 2.** Mediation analysis for homocysteine levels (*n*=1020) and niacin metabolites (*n*=250) | | | | | | | |
| --- | --- | --- | --- | --- | --- | --- | --- |
| Mediation investigated |  | Exposure | Dependent | Beta exposure | SE exposure | P-value | Model |
|  |  |  |  |  |  |  |  |
| 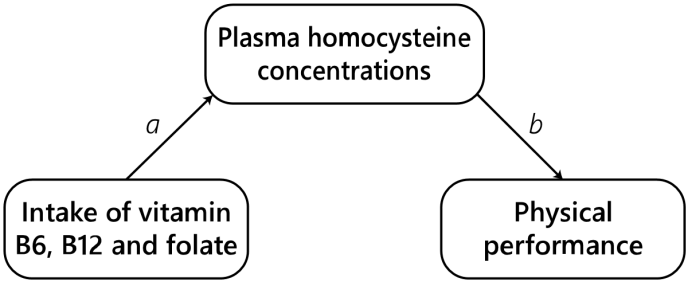 | *a* | B6, B12 and folate | Homocysteine | -0.43 | 0.14 | 0.003 | 3 |
|  |  | B6 | Homocysteine | -0.46 | 0.26 | 0.084 | 3 |
|  |  | B12 | Homocysteine | -0.003 | 0.001 | 0.056 | 3 |
|  |  | Folate | Homocysteine | -0.08 | 0.03 | 0.024 | 3 |
|  |  |  |  |  |  |  |  |
|  | *b* | Homocysteine | Handgrip strength | 0.03 | 0.04 | 0.446 | 3 |
|  |  | Homocysteine | Walk time (log) | 0.002 | 0.002 | 0.149 | 3 |
|  |  | Homocysteine | Chair rise test time (log) | 0.0004 | 0.002 | 0.843 | 3 |
|  |  |  |  |  |  |  |  |
| 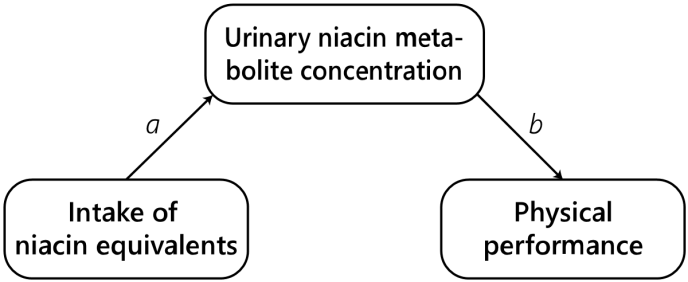 |  |  |  |  |  |  |  |
|  | *a* | Niacin equivalents | Niacin metabolites | 0.53 | 0.26 | 0.045 | 2 |
|  |  | Vitamin B3 | Niacin metabolites | 0.48 | 0.33 | 0.147 | 2 |
|  |  |  |  |  |  |  |  |
|  | *b* | Niacin metabolites | Handgrip strength | -0.008 | 0.018 | 0.649 | 2 |
|  |  | Niacin metabolites | Walk time (log) | 0.0004 | 0.0006 | 0.529 | 2 |
|  |  | Niacin metabolites | Chair rise test time (log) | -0.0007 | 0.0008 | 0.419 | 2 |
| Model 1. Adjusted for age and sex.  Model 2. Additional adjustment for energy intake, drinking status, smoking status, education level and physical activity level.  Model 3. Additional adjustment for study center and protein intake.  Model 2 was used for niacin equivalents / metabolites, as these data were only available from one study center, and protein intake is part of the calculation of niacin equivalents. | | | | | | | |

| **Supplemental Table 3.** Association between intake of the four different vitamins and measures of physical functioning with the exclusion of *n*=28 possible energy miss reporters (*n*=1209). | | | | | | | | |
| --- | --- | --- | --- | --- | --- | --- | --- | --- |
|  | Handgrip strength | | | | Walk time (log) | | Repeated chair rise time (log) | |
|  | β exposure | P-value | β interaction | P-value | β exposure | P-value | β of exposure | P-value |
|  |  |  | exposure * PASE-score | |  |  |  |  |
| **Niacin** |  |  |  |  |  |  |  |  |
| Model 1 | 0.07 ± 0.03 | 0.004 |  |  | -0.002 ± 0.001 | 0.053 | -0.001 ± 0.001 | 0.578 |
| Model 2 | 0.17 ± 0.07 | 0.010 | -0.001 ± 0.0004 | 0.048 | -0.002 ± 0.001 | 0.064 | -0.0002 ± 0.001 | 0.854 |
| Model 3 | 0.15 ± 0.06 | 0.015 | -0.001 ± 0.0004 | 0.028 | 0.0001 ± 0.001 | 0.933 | -0.0004 ± 0.001 | 0.745 |
| **Vitamin B-6** |  |  |  |  |  |  |  |  |
| Model 1 | 1.37 ± 0.31 | <0.0001 |  |  | -0.04 ± 0.01 | <0.001 | -0.024 ± 0.01 | 0.067 |
| Model 2 | 2.67 ± 0.77 | 0.0005 | -0.012 ± 0.005 | 0.015 | -0.04 ± 0.01 | 0.001 | -0.016 ± 0.01 | 0.270 |
| Model 3 | 1.70 ± 0.75 | 0.024 | -0.014 ± 0.005 | 0.003 | -0.02 ± 0.01 | 0.075 | -0.031 ± 0.02 | 0.053 |
| **Folate** |  |  |  |  |  |  |  |  |
| Model 1 | 0.003 ± 0.001 | 0.097 |  |  | -0.0001 ± 0.0001 | 0.013 | -0.0001 ± 0.0001 | 0.136 |
| Model 2 | 0.001 ± 0.002 | 0.732 |  |  | -0.0001 ± 0.0001 | 0.037 | -0.0001 ± 0.0001 | 0.581 |
| Model 3 | -0.002 ± 0.002 | 0.237 |  |  | -0.0001 ± 0.0001 | 0.055 | -0.0001 ± 0.0001 | 0.284 |
| **Vitamin B-12** |  |  |  |  |  |  |  |  |
| Model 1 | 0.151 ± 0.05 | 0.001 |  |  | -0.002 ± 0.002 | 0.229 | -0.002 ± 0.002 | 0.416 |
| Model 2 | 0.108 ± 0.05 | 0.028 |  |  | -0.001 ± 0.002 | 0.435 | -0.0002 ± 0.002 | 0.916 |
| Model 3 | -0.008 ± 0.05 | 0.870 |  |  | -0.001 ± 0.002 | 0.482 | -0.002 ± 0.002 | 0.359 |
| Model 1. Adjusted for age and sex. | | | | | | | | |
| Model 2. Additional adjustment for energy intake, drinking status, smoking status, education level and physical activity level. | | | | | | | | |
| Model 3. Additional adjustment for study center and protein intake. | | | | | | | | |
| PASE-score, Score on the Physical Activity Scale for the Elderly. | | | | | | | | |

| **Supplemental Table 4.** Association between intake of the four different vitamins and measures of physical functioning with the exclusion of *n*=240 B-supplement users (*n*=1057). | | | | | | | | |
| --- | --- | --- | --- | --- | --- | --- | --- | --- |
|  | Handgrip strength | | | | Walk time (log) | | Repeated chair rise time (log) | |
|  | β exposure | P-value | β interaction | P-value | β exposure | P-value | β of exposure | P-value |
|  |  |  | exposure * PASE-score | |  |  |  |  |
| **Niacin** |  |  |  |  |  |  |  |  |
| Model 1 | 0.09 ± 0.03 | 0.002 |  |  | -0.002 ± 0.001 | 0.040 | -0.0001 ± 0.001 | 0.884 |
| Model 2 | 0.20 ± 0.07 | 0.008 | -0.001 ± 0.001 | 0.062 | -0.002 ± 0.001 | 0.051 | -0.0003 ± 0.001 | 0.809 |
| Model 3 | 0.17 ± 0.07 | 0.020 | -0.001 ± 0.001 | 0.067 | -0.0003 ± 0.001 | 0.766 | -0.0005 ± 0.001 | 0.700 |
| **Vitamin B-6** |  |  |  |  |  |  |  |  |
| Model 1 | 1.55 ± 0.35 | <0.0001 |  |  | -0.05 ± 0.01 | <0.0001 | -0.027 ± 0.014 | 0.059 |
| Model 2 | 2.83 ± 0.88 | 0.0013 | -0.011 ± 0.006 | 0.043 | -0.05 ± 0.01 | 0.0001 | -0.013 ± 0.016 | 0.413 |
| Model 3 | 1.76 ± 0.87 | 0.042 | -0.013 ± 0.005 | 0.017 | -0.04 ± 0.02 | 0.011 | -0.024 ± 0.018 | 0.182 |
| **Folate** |  |  |  |  |  |  |  |  |
| Model 1 | 0.003 ± 0.002 | 0.086 |  |  | -0.0002 ± 0.0001 | 0.003 | -0.0001 ± 0.0001 | 0.125 |
| Model 2 | 0.002 ± 0.002 | 0.455 |  |  | -0.0002 ± 0.0001 | 0.013 | -0.0001 ± 0.0001 | 0.689 |
| Model 3 | -0.001 ± 0.002 | 0.621 |  |  | -0.0002 ± 0.0001 | 0.012 | -0.0001 ± 0.0001 | 0.380 |
| **Vitamin B-12** |  |  |  |  |  |  |  |  |
| Model 1 | 0.141 ± 0.05 | 0.008 |  |  | -0.002 ± 0.002 | 0.369 | -0.003 ± 0.002 | 0.155 |
| Model 2 | 0.094 ± 0.05 | 0.077 |  |  | -0.001 ± 0.002 | 0.700 | -0.001 ± 0.002 | 0.581 |
| Model 3 | -0.012 ± 0.06 | 0.827 |  |  | -0.001 ± 0.002 | 0.761 | -0.003 ± 0.002 | 0.231 |
| Model 1. Adjusted for age and sex. | | | | | | | | |
| Model 2. Additional adjustment for energy intake, drinking status, smoking status, education level and physical activity level. | | | | | | | | |
| Model 3. Additional adjustment for study center and protein intake. | | | | | | | | |
| PASE-score, Score on the Physical Activity Scale for the Elderly. | | | | | | | | |


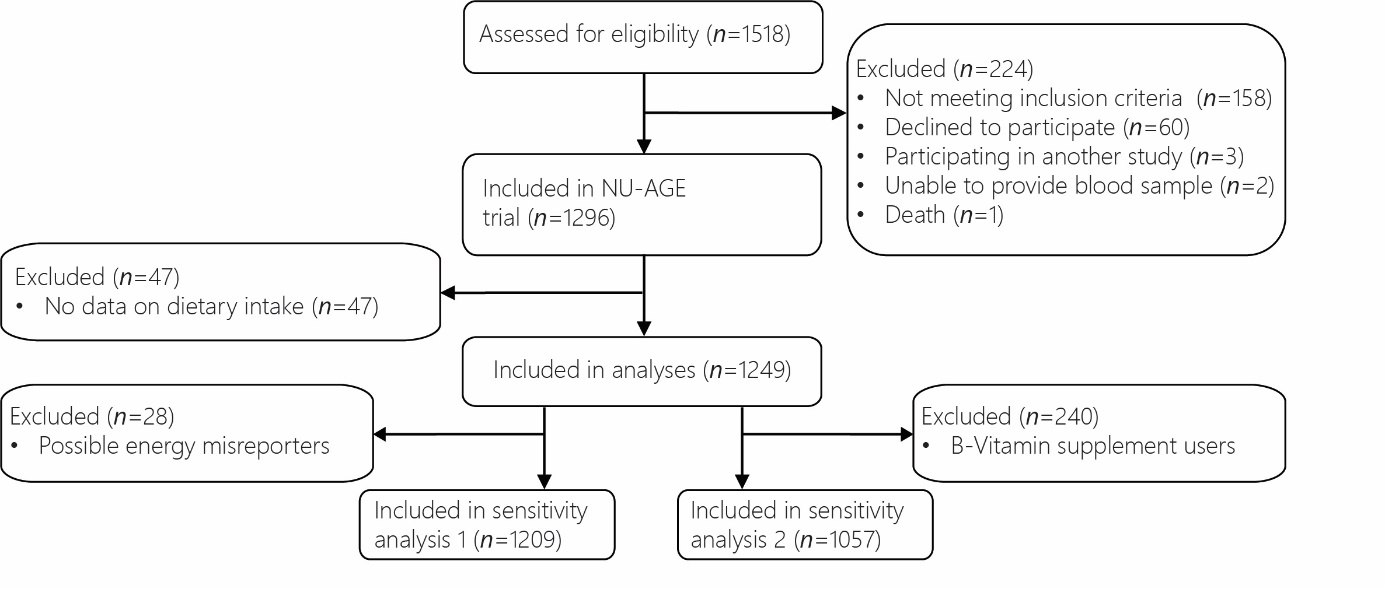


**Supplemental Figure 1**. Flowchart of participants
